# Astrocytes and Müller Cell Alterations During Retinal Degeneration in a Transgenic Rat Model of Retinitis Pigmentosa

**DOI:** 10.3389/fncel.2015.00484

**Published:** 2015-12-22

**Authors:** Laura Fernández-Sánchez, Pedro Lax, Laura Campello, Isabel Pinilla, Nicolás Cuenca

**Affiliations:** ^1^Department of Physiology, Genetics and Microbiology, University of AlicanteAlicante, Spain; ^2^Department of Ophthalmology, Aragon Institute for Health Research, Lozano Blesa University HospitalZaragoza, Spain; ^3^Institute Ramón Margalef, University of AlicanteAlicante, Spain

**Keywords:** astrocytes, müller cells, gliosis, immunolabeling, P23H, retinal remodeling

## Abstract

**Purpose:** Retinitis pigmentosa includes a group of progressive retinal degenerative diseases that affect the structure and function of photoreceptors. Secondarily to the loss of photoreceptors, there is a reduction in retinal vascularization, which seems to influence the cellular degenerative process. Retinal macroglial cells, astrocytes, and Müller cells provide support for retinal neurons and are fundamental for maintaining normal retinal function. The aim of this study was to investigate the evolution of macroglial changes during retinal degeneration in P23H rats.

**Methods:** Homozygous P23H line-3 rats aged from P18 to 18 months were used to study the evolution of the disease, and SD rats were used as controls. Immunolabeling with antibodies against GFAP, vimentin, and transducin were used to visualize macroglial cells and cone photoreceptors.

**Results:** In P23H rats, increased GFAP labeling in Müller cells was observed as an early indicator of retinal gliosis. At 4 and 12 months of age, the apical processes of Müller cells in P23H rats clustered in firework-like structures, which were associated with ring-like shaped areas of cone degeneration in the outer nuclear layer. These structures were not observed at 16 months of age. The number of astrocytes was higher in P23H rats than in the SD matched controls at 4 and 12 months of age, supporting the idea of astrocyte proliferation. As the disease progressed, astrocytes exhibited a deteriorated morphology and marked hypertrophy. The increase in the complexity of the astrocytic processes correlated with greater connexin 43 expression and higher density of connexin 43 immunoreactive puncta within the ganglion cell layer (GCL) of P23H vs. SD rat retinas.

**Conclusions:** In the P23H rat model of retinitis pigmentosa, the loss of photoreceptors triggers major changes in the number and morphology of glial cells affecting the inner retina.

## Introduction

Retinal macroglia, consisting of astrocytes and Müller cells, play key roles in the homeostasis of retinal neurons, keeping the retina healthy, and functioning properly. Müller cells, the largest glial cells in the retina of vertebrates, cover practically the entire retinal thickness (from the outer to the inner limiting membrane) and make contact with both neuronal somata and processes in the entire retina. Müller glial cells are thought to play an essential role in maintaining the structural integrity of the retina and to participate in essential processes, such as glucose metabolism, neurotransmitter uptake, and retinal homeostasis (Reichenbach and Bringmann, 2013; Chong and Martin, 2015). Astrocytes, almost entirely restricted to the retinal nerve fiber layer, have a close relationship with neurons and the major blood vessels. They are commonly thought to play an important part in the proper development and functioning of the vascular system in the retina, including blood flow and the formation of the blood-retinal barrier (BRB) (Coorey et al., 2012; Kur et al., 2012; Klaassen et al., 2013). Both astrocytes and Müller cells are involved in the survival of retinal cells through the release of neurotrophic factors, providing anti-oxidative support, clearing neurotransmitters and ions from the extraneural space and, as in the brain, supporting the formation and removal of synapses. They are also involved in the activation of microglial cells and participate in the regulatory mechanisms of vasodilation and vasoconstriction (Azevedo et al., 2009; Bringmann and Wiedemann, 2012; Coorey et al., 2012; Bringmann et al., 2013; Cuenca et al., 2014; Chong and Martin, 2015; Vecino et al., 2015).

Astrocyte activation and reactive gliosis are common traits in neurodegenerative processes. A hallmark of gliosis is the upregulation of intermediate filament proteins in glial cells, including glial fibrillary acidic protein (GFAP), vimentin, and nestin (Anderson et al., 2008; Luna et al., 2010). Increasing evidence points to both the benefits and adverse effects that reactive gliosis can have on already injured neurons. The neuroprotective effects of activated glial cells include the production of neurotrophic factors, growth factors, and cytokines (Harada et al., 2003; Bringmann et al., 2006), whereas the dysfunction of glial cells in different pathologies of the retina has been linked to retinal swelling and BRB breakdown (Shen et al., 2010; Klaassen et al., 2013). In contrast, chronic gliosis might accelerate neurodegeneration over the course of a chronic illness, resulting in both direct and indirect damage to neurons and the vascular system in the retina. In this context, chronic gliosis exacerbates the progression of the disease, making vessels more permeable, and enhancing the infiltration of toxic compounds (Bringmann et al., 2006; Coorey et al., 2012). Furthermore, in mature retinas, astrocytes, and Müller cells play a role in the neovascularization linked to pathological processes, such as age-related macular degeneration (AMD), diabetic retinopathy (DR), and retinopathy of prematurity (ROP), as they have been shown to release angiogenic growth factors in the presence of pathogenic stimuli (Penn et al., 2008).

Retinitis pigmentosa (RP) is a heterogeneous group of retinal degenerative disorders with a polymorphic hereditary origin that cause a progressive loss of retinal function, and it represents a major cause of blindness. Approximately 20–25% of autosomal dominant RP patients exhibit a mutation in the rhodopsin gene, with P23H being one of the most common rhodopsin mutations (Dryja et al., 1990), accounting for about one-third of such cases in the USA (Dryja et al., 2000). The P23H mutation is now known to provoke the retention and misfolding of rhodopsin in the endoplasmic reticulum (Kaushal and Khorana, 1994). Some studies have also pointed to a mechanism for RP in which cellular stress triggers an inflammatory response, which is followed by retinal remodeling, and the final common pathway of programmed photoreceptor cell death, i.e., apoptosis (Remé et al., 1998; Illing et al., 2002; Marc et al., 2003; Jones et al., 2012; Cuenca et al., 2014). Similar mechanisms have been found in other forms of retinal degeneration, such as glaucoma, DR, and AMD (Cuenca et al., 2014).

Transgenic P23H rats have been engineered to mimic human P23H RP (Dryja et al., 1990, 2000). Although they initially exhibit normal cone function, these rats develop a progressive rod dysfunction that is consistent in broad terms with the clinical findings reported for human patients with P23H RP (Berson et al., 1991; Machida et al., 2000; Cuenca et al., 2004; Pinilla et al., 2005). Photoreceptor loss has been observed to accompany degeneration of the inner retina (Marc et al., 2003; Cuenca et al., 2004, 2014; Jones and Marc, 2005; Puthussery and Taylor, 2010; Jones et al., 2012), in addition to considerable degeneration of retinal ganglion cells (Jones et al., 2003; García-Ayuso et al., 2010; Kolomiets et al., 2010).

Considering the key role of glial cells in maintaining the structure, function, and survival of retinal neurons, it is important to expand our understanding of the cellular changes in glial cells associated with retinal injuries and neurodegenerative diseases. The aim of this study was to investigate the evolution of macroglial changes during retinal degeneration in RP and to evaluate the role of macroglia in inner retina remodeling after photoreceptor death. This study uses albino P23H rats at different ages as animal models of RP.

## Materials and methods

### Animals

Homozygous albino P23H line three rats (*n* = 26) obtained from Dr. M. LaVail (UCSF) were used as a model of RP. Normal SD rats (*n* = 26) obtained from Harlan Laboratories (Barcelona, Spain) were used as wild-type controls. All animals were bred in a colony at the University of Alicante, Spain, and maintained under controlled humidity (60%), temperature (23 ± 1°C) and photoperiod (LD 12:12) conditions. Light was provided by two fluorescent lamps, with an intensity of 350–400 lux at cage level. Dry food and water were made available *ad libitum*. All animals were housed, handled and the procedures carried out according to Project License UA-2013-07-22, approved by the Ethic Committee for Animal Experiment from the University of Alicante. All procedures were performed according to current regulations regarding the use of laboratory animals (NIH, ARVO, and European Directive 2010/63/UE), which are intended to limit both animal suffering and the number of animals required for experimentation.

### Retinal histology

#### Retinal sections

Animals were sacrificed in the morning, between 10:00 a.m. and 12:00 p.m., by administering a lethal dose of pentobarbital. After marking the dorsal margin of the limbus with a stitch, the eyes were enucleated, fixed in 4% (w/v) paraformaldehyde 1 h at room temperature (RT), washed and then sequentially cryoprotected in 15, 20, and 30% sucrose. The cornea, lens, and vitreous body were removed, and the eyecups were processed for vertical sections or whole mounts. For cryostat sections, eyecups were frozen in OCT with liquid nitrogen. Fourteen-micrometer-thick sections were then obtained using a Leica CM 1900 cryostat (Leica Microsystems, Wetzlar, Germany) mounted on Superfrost Plus slides (Menzel GmbH & Co KG, Braunschweig, Germany) and air-dried. Before further processing, the slides were washed three times in phosphate buffer (PB), and then treated with blocking solution (10% normal donkey serum in PB plus 0.5% TritonX-100) for 1 h.

#### Retinal immunofluorescence

In order to objectively compare them, retinas from P23H and SD rats were fully processed in parallel. All primary antibodies and lectins used in this work (summarized in Table 1) have been previously used in other studies and have been well characterized by us and other authors with regard to the specific cell type molecular markers. Sections and whole mount retinas were either single- or double-immunostained at room temperature overnight or for 3 days, respectively, using combinations of antibodies targeting distinct molecular markers. The dilutions in PB with 0.5% Triton X-100 are indicated in Table 1. Afterwards, Alexa Fluor 488 (green)-conjugated anti-rabbit IgG and/or Alexa Fluor 555 (red)-conjugated anti-mouse IgG donkey secondary antibodies from Molecular Probes (Eugene, OR) were added at a dilution rate of 1:100 for 1 h for sections and overnight for whole mount retinas. TO-PRO-3 iodide (Molecular Probes) was used as a nuclear marker. Finally, the retinas were washed in PB, mounted in Citifluor (Citifluor Ltd, London, UK) and coverslipped for viewing with laser-scanning confocal microscopy on a Leica TCS SP2 system (Leica Microsystems). Immunohistochemical controls were carried out by omitting either the primary or secondary antibodies. The resulting images from the control and experimental subjects were processed in parallel with Adobe Photoshop 10.0 software (Adobe Systems Inc, San José, CA, USA).

**Table 1:** **Primary antibodies and lectins used in this work**.

**Molecular marker (initials)**	**Antibody/lectin**	**Company**	**Catalog ref**.	**Working dilution**	
				**IHC**	**WB**
Glial fibrillary acidic protein (GFAP)	Mouse, G-A-5	Sigma	G 3893	1:500	–
Glial fibrillary acidic protein (GFAP)	Rabbit, polyclonal	Dako	N1506	1:50	1:1000
Transducin, Gαc subunit (Gt)	Rabbit, polyclonal	Cytosignal	PAB-00801-G	1:200	–
Vimentin	Mouse, V9	DAKOCytomation	M0725	1:100	–
Connexin 43 (Cx43)	Rabbit, polyclonal	Sigma	C 6219	1:1000	1:10000
*Griffonia simplicifolia* IB_4_ (GS-IB_4_)	Isolectin IB_4_	Invitrogen	I21411	1:100	–
Collagen type IV	Goat, polyclonal	Millipore	AB769	1:100	–
Glyceraldehyde-3-phosphate dehydrogenase (GAPDH)	Mouse, 6C5	Millipore	MAB374	–	1:10000

Primary antibodies and lectins used in this work. The two first columns list the antigen and the animal origin of the antibody. The clone code is specified for monoclonal antibodies. The two central columns provide the commercial company and antibody catalog reference. The two last columns specify the dilution at which each antibody was used for immunohistochemistry (IHC) or immunoblotting (WB).

#### Astrocyte quantification

Astrocytes were quantified on whole-mount retinas at different ages. Immunolabeling against GFAP and the nuclear marker TO-PRO-3 were used to identify astrocytes. Lectin was used to visualize blood vessels. Twelve representative regions, each measuring 0.227 mm^2^, were analyzed from each retina: six equidistant regions on the superior-inferior axis and six fields on the temporal-nasal axis. This ensured representative sampling of the peripheral, medial and central zones of the upper, lower, temporal, and nasal quadrants of each retina. In all 12 regions, each astrocyte cell body was manually counted in two adjacent confocal images and the cell density values were averaged. To avoid overestimation of astrocyte density, only GFAP-positive cells with a well-defined nucleus were included in the count. At least three rats were used for each experimental group.

### Immunoblotting

GFAP and connexin 43 protein expression was assessed using Western blotting on P23H and SD rat retinas at 4 and 12 months of age (four rats per experimental group). Briefly, proteins were extracted and subjected to immunoblotting analysis. Proteins (30 μg/lane) were resolved by SDS-PAGE on 12% polyacrylamide gels and electrotransferred to nitrocellulose membranes (GE Healthcare, Buckinghamshire, UK). Membranes were blocked in 5% bovine serum albumin (BSA) diluted in 25 mM Tris (pH 8.0), 150 mM NaCl, 2.7 mM KCl (TBS) with 0.1% v/v Tween 20 for 2 h. Afterwards, membranes were probed at 4°C overnight with rabbit polyclonal GFAP or connexin 43 antibodies diluted in TBS, and with mouse monoclonal GAPDH antibodies (see working dilutions on Table 1). After incubation with primary antibodies, the membranes were incubated at room temperature for 1 h with horseradish peroxidase-conjugated goat anti-rabbit or goat anti-mouse (Pierce, Rockford, IL, USA) IgG at a 1:10,000 dilution. Detection was performed by enhanced chemiluminescence using the SuperSignal West Dura system (Pierce).

Western blot films were digitized using a scanner in transparency mode at 300 dpi and 16 bits gray scale. Densitometric quantitation of protein bands was accomplished using the Quantity One software from BioRad (Hercules, CA, USA), and the values obtained for each protein were normalized to GAPDH levels. For each quantified protein, we analyzed the results of at least two independent experiments.

### Statistical analysis

A Two-way ANOVA was used to evaluate the effects of genotype (SD vs. P23H) and age (P18 to 16 months), alone and in combination. When the level of significance was 0.05 or less, *post-hoc* pairwise comparisons using Bonferroni's test were performed. Normal distribution and homogeneity of variance were determined for the categories of the previously defined variables. Data are reported as mean ± SEM. Values of *P* < 0.05 were considered to be statistically significant. All statistical analyses were performed using SPSS 15.0 software (Statistical Package for Social Sciences, Chicago, IL, USA). All counts were made by two trained observers, using a blind methodology.

## Results

### Astrocyte morphology and distribution in normal and degenerating rat retinas

**Figure 1:**
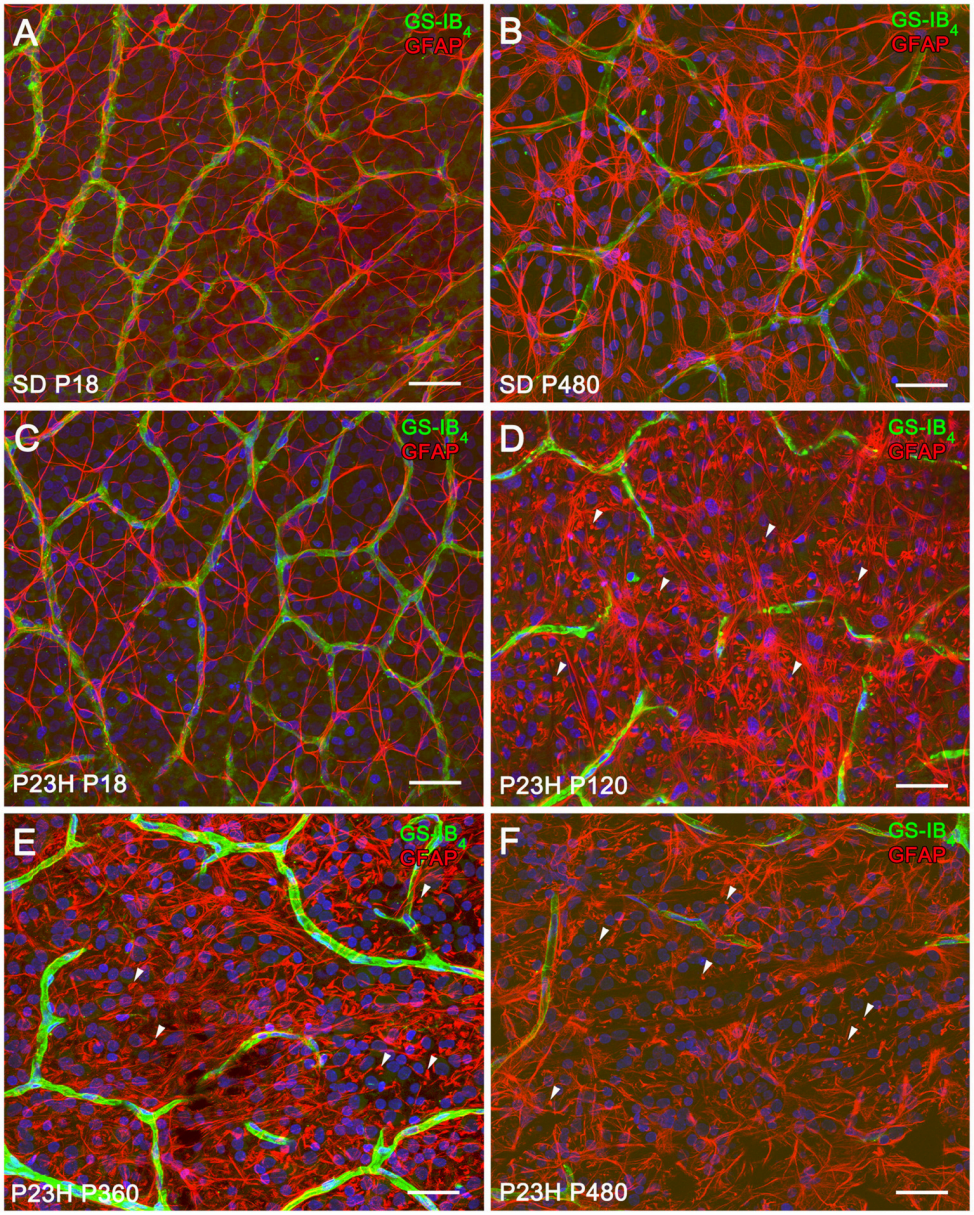
**Distribution and density of astrocytes in SD and P23H rat retinas**. **(A–F)** Whole-mounted retinas from SD **(A,B)** and P23H rats **(C–F)** at P18 **(A,C)**, P120 **(D)**, P360 **(E)**, and P480 **(B,F)** showing retinal astrocytes (red) at the nerve fiber layer and blood vessels (green). Nuclei were stained with the nuclear marker TO-PRO 3 (blue). Astrocytes have been labeled with antibodies against GFAP. Blood vessels have been labeled with *Griffonia simplicifolia* isolectin B4. All images were collected from the medial area of the retina. Note the presence of GFAP positive Müller cell end-feet in P23H rats at P120 (**D**, arrowheads), P360 (**E**, arrowheads), and P480 (**F**, arrowheads). Scale bar: 40 μm.

P23H rat retinas undergo neurodegenerative changes associated with the progression of the disease. To assess the evolution of astrocyte changes during retinal degeneration, the cell density and morphology of retinal astrocytes were studied at different ages in normal and diseased retinas through the immunocytochemical localization of GFAP. Figure 1 illustrates the distribution and cell density of astrocytes in whole-mounted retinas from SD (Figures 1A,B) and P23H (Figures 1C–F) rats. In normal and diseased retinas, astrocytes were distributed in a plexus located on the ganglion cell layer (GCL). In P23H rat retinas, GFAP-positive Müller cell end-feet were observed at the GCL from P120 to P480 (Figures 1D–F, arrowheads).

**Figure 2:**
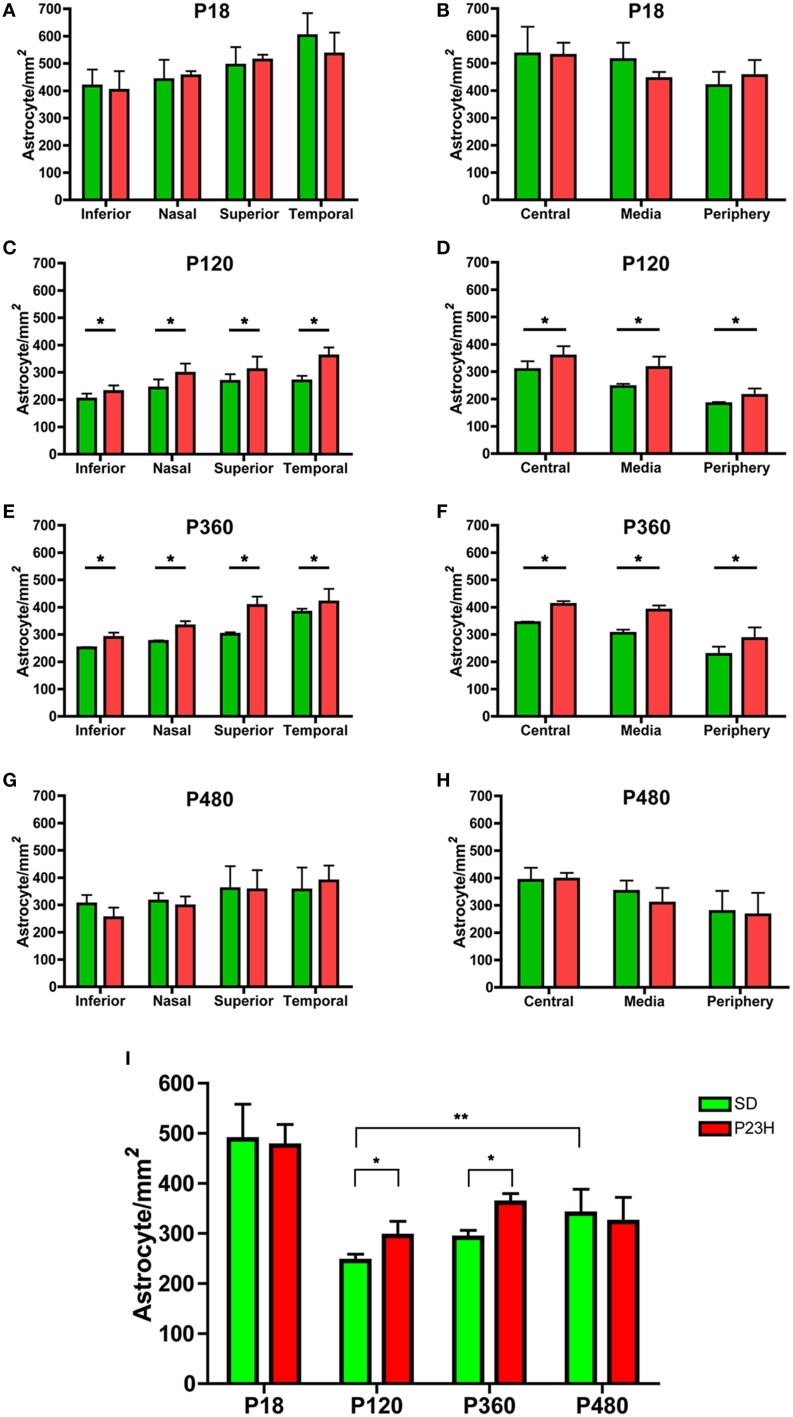
**Astrocyte density in SD and P23H rat retinas**. Quantification of the cell density of astrocytes in whole-mounted retinas from SD (green) and P23H rats (red). Astrocytes were quantified on representative sampling of the peripheral, medial, and central zones of the upper, lower, temporal, and nasal quadrants of each retina at P18 **(A,B)**, P120 **(C,D)**, P360 **(E,F)**, and P480 **(G,H)**. Mean density values for all representative samplings are represented in **(I)**. Data are reported as mean ± SEM. ^*^P < 0.05; ^**^P < 0.01.

Astrocyte quantification showed that astrocyte density gradually increased from the periphery toward the central area of the retina in both SD and P23H rats at all ages analyzed (Figures 2B,D,F,H). In addition, the astrocyte density was higher in the temporal and superior areas of the retina (Figures 2A,C,E,G). In both SD and P23H rats, astrocyte density was significantly lower in adults as compared to P18 aged rats (ANOVA, Bonferroni's test, *P* < 0.001; Figure 2I), and the mean density of astrocytes increased slightly from P120 to P480 in both SD and P23H rats, with significant differences between P120 and P480 SD rats (ANOVA, Bonferroni's test, *P* < 0.01). On the other hand, a significant increase in astrocyte cell numbers was found in P120 and P360 P23H rat retinas, as compared to control SD rats (ANOVA, Bonferroni's test, *P* < 0.05; Figure 2I), with significant differences in all retinal regions analyzed (ANOVA, Bonferroni's test, *P* < 0.05; Figures 2C–F).

**Figure 3:**
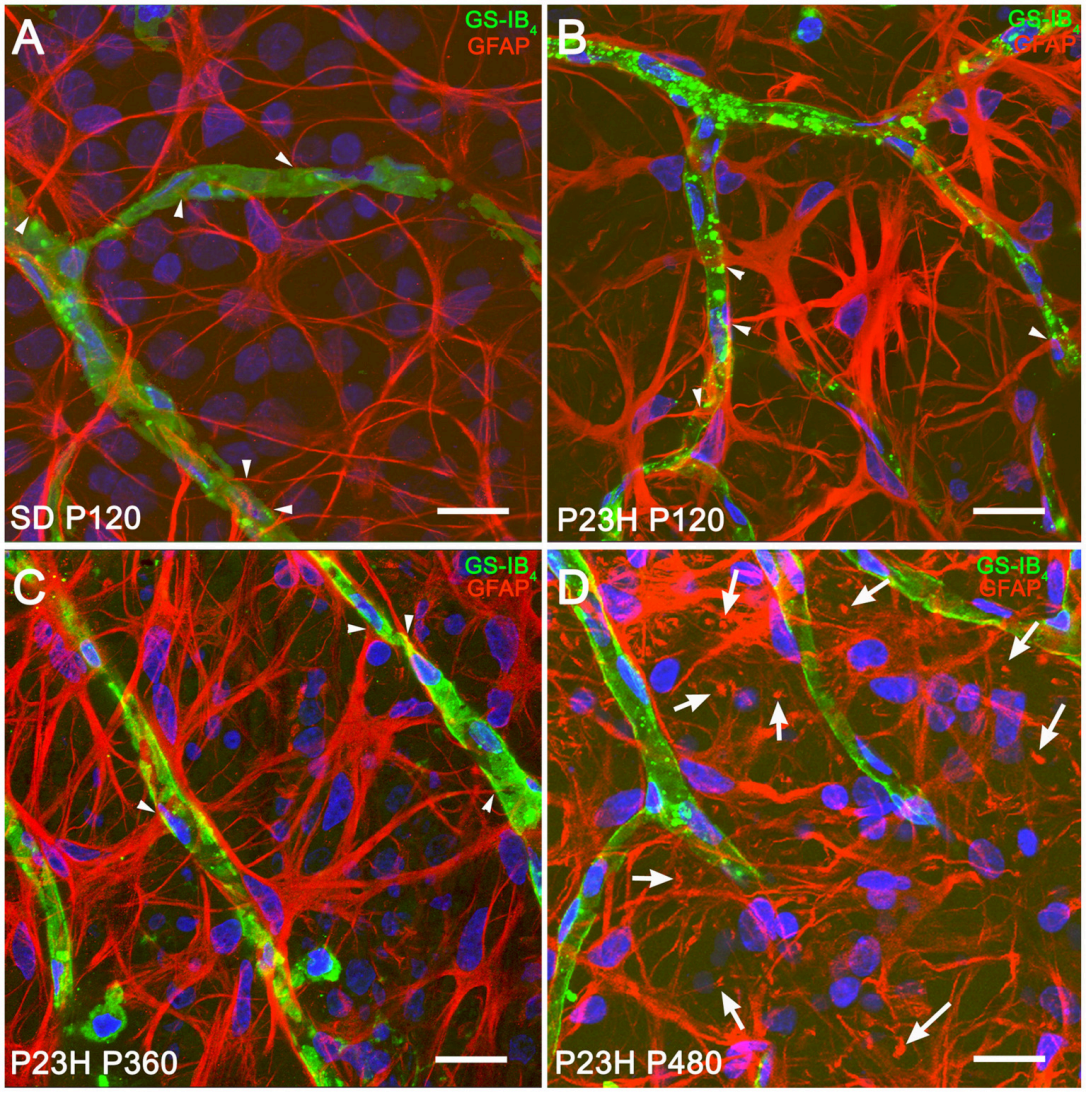
**Morphological and structural features of astrocytes in SD and P23H rat retinas**. Whole-mounted retinas from a P120 SD rat **(A)** and P23H rats at P120 **(B)**, P360 **(C)**, and P480 **(D)**, showing retinal astrocytes (red) at the nerve fiber layer and blood vessels (green). Nuclei were stained with the nuclear marker TO-PRO 3 (blue). Astrocytes have been labeled with antibodies against GFAP. Blood vessels have been labeled with *G. simplicifolia* isolectin B4. All images were collected from the medial area of the retina. Note the presence of GFAP positive Müller cell end-feet in P23H rats at P480 (arrows). Scale bar: 20 μm.

In both normal and diseased retinas, astrocytes showed the characteristic morphology, with flattened cell body and a series of fibrous radiating processes. As illustrated in Figure 3, astrocyte processes, and even occasionally, astrocyte cell bodies were intimately associated with the blood vessels of the superficial retinal vascular plexus, with astrocyte end-feet encircling endothelial cells (Figures 3A–C, arrowheads). In P23H rats, astrocytic hyperplasia was associated to astrocyte process hypertrophy accompanied by increased GFAP immunoreactivity. Interactions between astrocyte cell bodies and blood vessels were less evident (Figure 3D) in more advanced stages of degeneration (P480).

**Figure 4:**
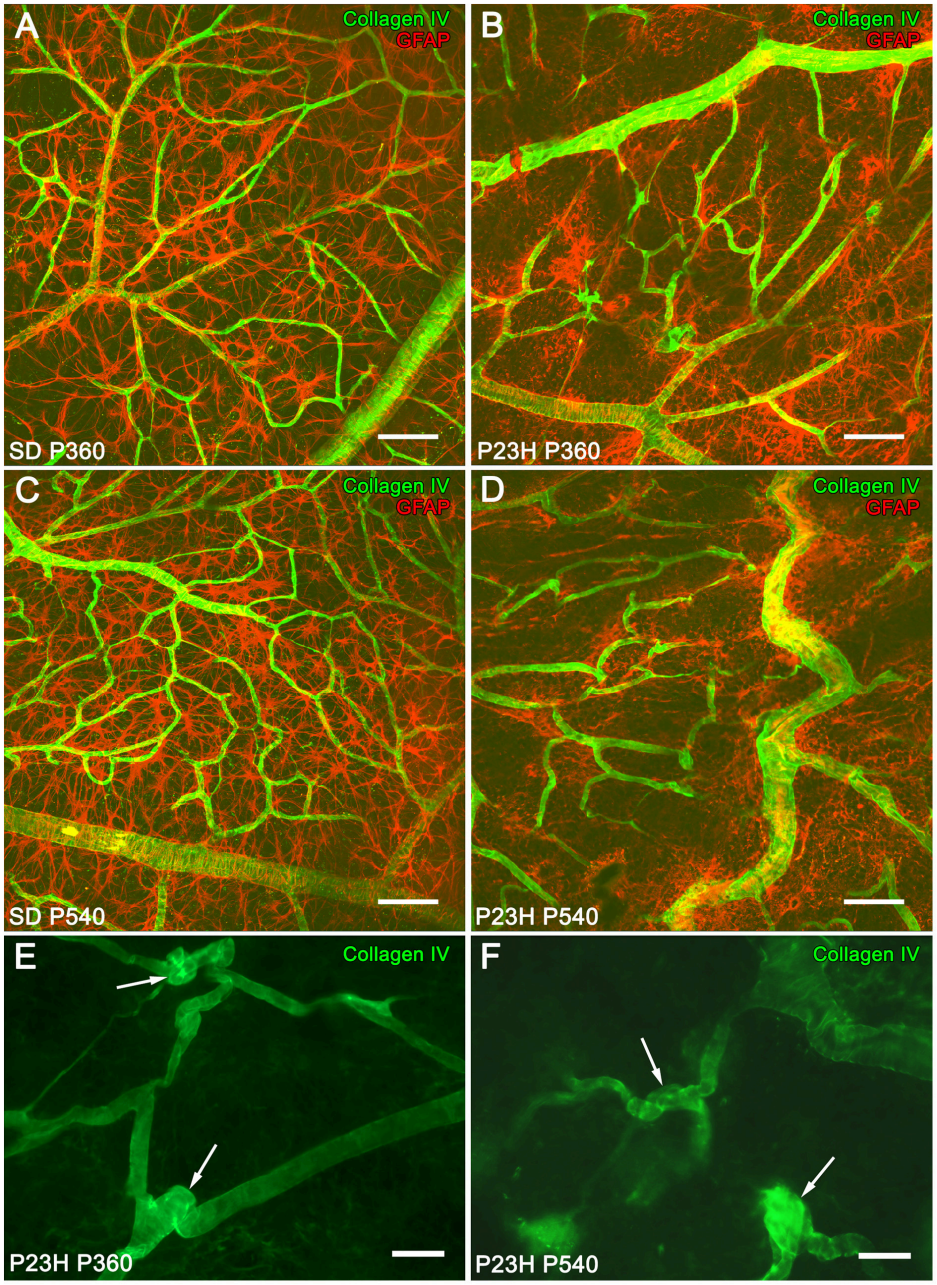
**Superficial vascular plexus in SD and P23H rat retinas**. Whole-mounted retinas from SD rats **(A,C)** and P23H rats **(B,D–F)** at P360 **(A,B,E)**, and P540 **(C,D,F)**, stained for collagen IV (green) and GFAP (red) in order to show the relationship between blood vessels and astrocytes, respectively, during retinal degeneration. During aging, blood vessels in P23H rat retinas **(B,D)** suffered alterations related to astrocyte changes that were unobserved in SD rats **(A,C)**. Note the formation of blood vessel tangles at P360 (**E**, arrows) and P540 (**F**, arrows). Scale bar: 100 μm **(A,D)**, 40 μm **(E,F)**.

In 12- and 18-month-old SD rat retinas, astrocytes with normal morphology and distribution were closely associated with typical retinal blood vessels (Figures 4A,C). However, at these ages, P23H rats showed a progressive disruption of the superficial vascular plexus, exhibiting tortuous vessels (Figures 4B,D). Blood vessels were significantly regressed, and loops largely degenerated. Moreover, P23H rat retinas showed the formation of blood vessel tangles at 12 (Figure 4E, arrows) and 18 (Figure 4F, arrows) months of age. Figures 4B,D show that superficial vascular plexus degeneration was associated with the loss of normal morphology in astrocyte cells.

### Increase of connexin 43 immunofluorescence in P23H rat retinas

**Figure 5:**
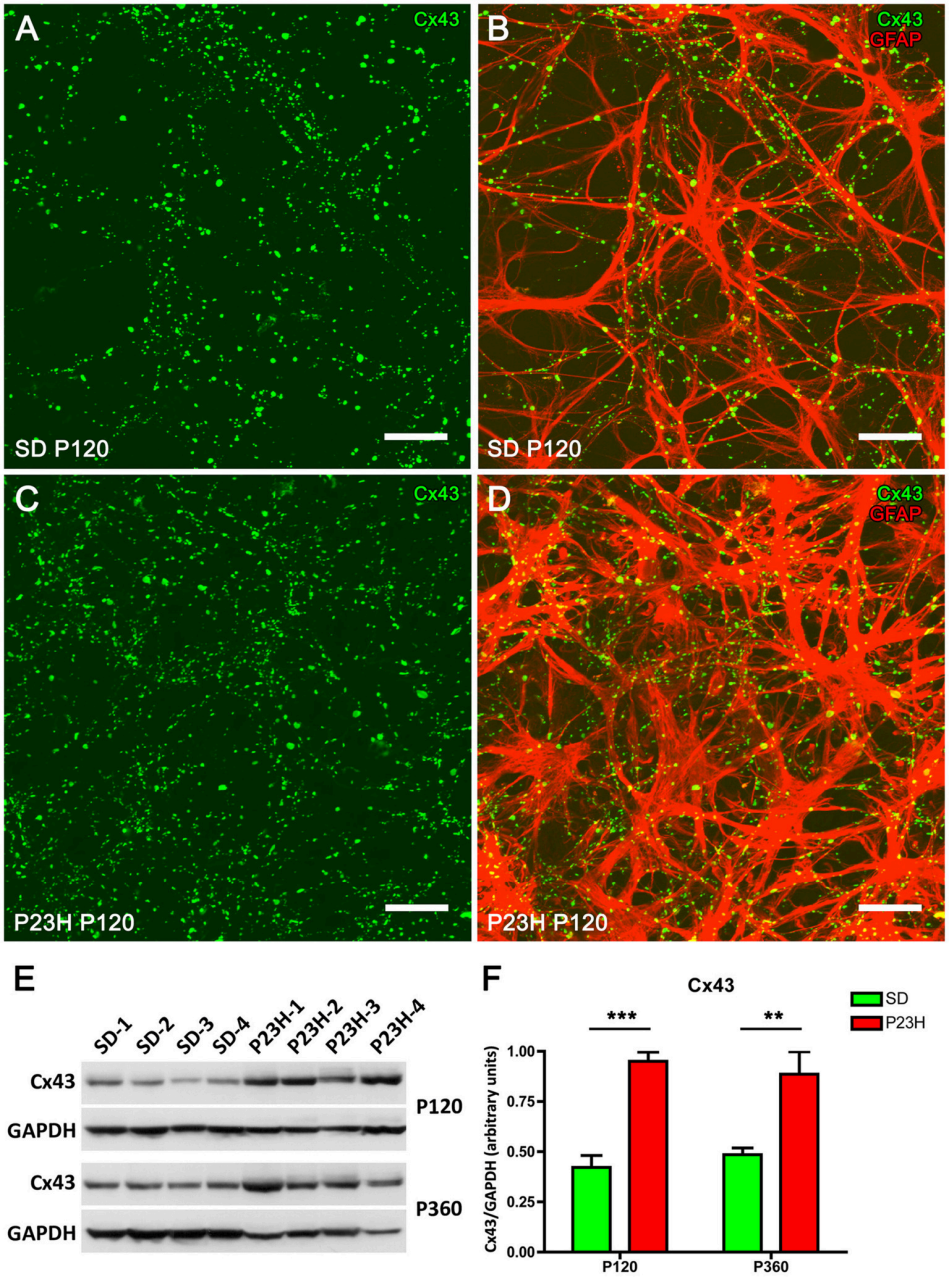
**Connexin 43 expression in SD and P23H rat retinas**. Whole-mounted retinas from a SD rat **(A,B)** and a P23H rat **(C,D)** at P120. Connexin 43 (Cx43, green) and GFAP immunolabeling (red) show the relationship between retinal astrocytes and Cx43-immunoreactive (Cx43-IR) puncta. Note that Cx43-IR puncta are located along GFAP-IR astrocyte processes **(B,D)** and are more numerous in P23H rat retinas **(C,D)**, as compared to the control SD rat retinas **(A,B)**. Scale bar: 20 μm. **(E)** Detection of Cx43 (43 kDa) in P23H rat retinas and SD controls by Western blotting at P120 and P360. GAPDH (36 kDa) levels are shown as loading controls. **(F)** The amount of Cx43 protein present in each band was densitometrically measured and the value obtained for each sample was normalized to GAPDH levels. Bars represent the average ± SEM (*n* = 4). ^**^P < 0.01; ^***^P < 0.001.

Connexin 43 (Cx43) is the most abundant gap junction protein in the central nervous system (CNS) including retina, and is expressed mainly on astrocyte processes surrounding blood vessels (Nagy and Rash, 2000; Zahs et al., 2003; Kerr et al., 2010). To determine the relationship between the expression of Cx43 and astrocytic hyperplasia and hypertrophy in the P23H rat retina, whole-mounted retinas from P120 SD and P23H rats were double-labeled with antibodies against Cx43 and GFAP. In both SD and P23H rats, Cx43-immunoreactive (IR) puncta were located along GFAP-IR astrocyte processes in the GCL (Figure 5). However, the density of Cx43-IR puncta within the GCL was greater in P23H (Figures 5C,D) vs. SD rat retinas (Figures 5A,B).

To quantify the differential expression of Cx43 gap junction proteins in normal and diseased retinas, we performed immunoblotting in 4- and 12-month-old rat retinas from SD and P23H rats (Figures 5E,F). A 43-kDa band corresponding to Cx43 protein was observed, which was more intense in P23H rat retinas than in SD controls (Figure 5E). Antibodies against GAPDH (36 kDa) were used as loading controls for Western blotting experiments. The amount of Cx43 protein present in each band was densitometrically measured and the value obtained for each sample was normalized to GAPDH levels. The normalized expression levels were significantly upregulated in the diseased rat retinas as compared to controls. Expression fold changes were calculated as the ratio between the P23H and control normalized values. In particular, Cx43 levels were found to be increased 2.25- and 1.81-fold in the 4- and 12-month-old P23H rat retinas, respectively, as compared to controls (ANOVA, Bonferroni's test, *P* < 0.001 in both cases).

### Increased Müller cell expression of vimentin and GFAP in P23H rat retinas

**Figure 6:**
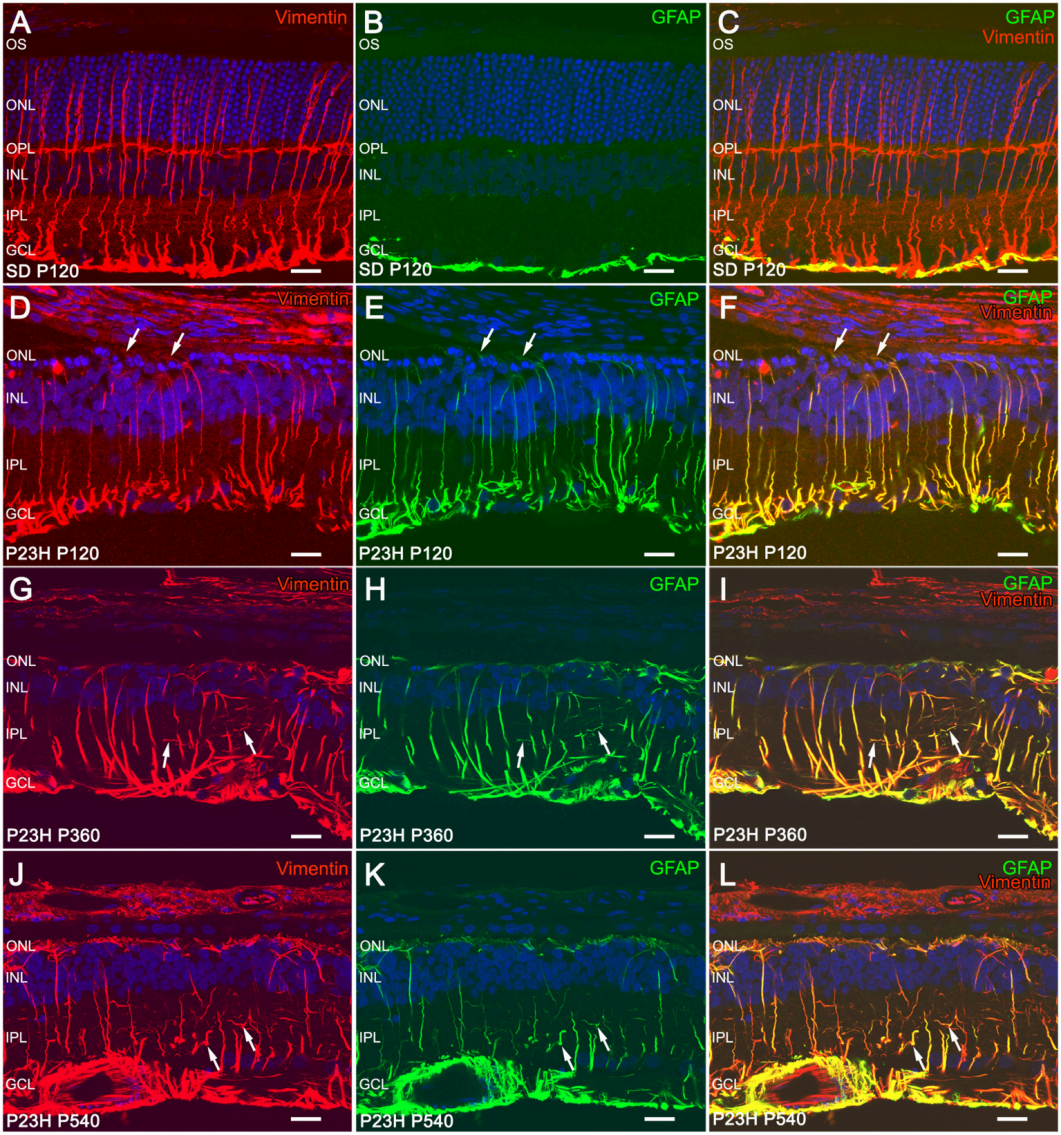
**Immunofluorescence of vimentin and GFAP in SD and P23H rat retinas**. Vertical retinal sections from P120 SD rats **(A–C)** and P23H rats at P120 **(A–F)**, P360 **(G–I)**, and P540 **(J–L)**. Nuclei were stained with the nuclear marker TO-PRO 3 (blue). Immunostaining with GFAP (green) and vimentin (red) shows reactive changes in Müller cells associated with retinal degeneration in P23H rats. All images were collected from the medial area of the retina. ONL, outer nuclear layer; OPL, outer plexiform layer; INL, inner nuclear layer; IPL, inner plexiform layer; GCL, ganglion cell layer. Scale bar: 20 μm.

GFAP expression in Müller cells is an indicator of tissue stress, and it has been associated with retinal degeneration, whereas the intermediate filament protein vimentin is ubiquitously expressed in the Müller cells of many mammalian species. Figure 6 shows the immunohistochemical localization of GFAP and vimentin in vertical retinal sections from SD (Figures 6A–C) and P23H (Figures 6D–L) rats. In Müller cells from normal retinas, vimentin immunostaining was distributed throughout the entire processes, from their end-feet up to the outer retina (Figure 6A), while GFAP immunoreactivity was virtually limited to the inner margin of the retina, colocalizing with astrocytes (Figure 6B). In fact, the colocalization of vimentin and GFAP was negligible in SD rats (Figure 6C).

In P23H rats, vimentin immunoreactivity within Müller cells was similar to that observed in age-matched control rats (Figures 6A,D,G,J). However, diseased retinas showed intense GFAP immunoreactivity in Müller cells, which exhibited a marked labeling throughout the entire cell (Figures 6E,H,K). Thus, double staining for vimentin and GFAP revealed extensive colocalization of both intermediate filament proteins in these rats (Figures 6F,I,L). Vimentin/GFAP-positive Müller cell apical processes extended into the subretinal space. These glial components expanded, filling areas previously occupied by degenerated photoreceptors (Figures 6D–F, arrows). In agreement with previous reports (Cuenca et al., 2004, 2014), 4-month-old P23H rats showed around two rows of photoreceptor cell bodies in the central area of the retina, whereas 4-month-old SD rats showed around 12 photoreceptor rows in the same area. At 18 months of age and thereafter, photoreceptor cells were virtually absent from P23H rat retinas (data not shown). At 12 and 18 months of age, Müller cells have lost their parallel arrangement at IPL level, and their lateral branches had become enlarged and chaotically entangled (Figures 6G–L, arrows).

**Figure 7:**
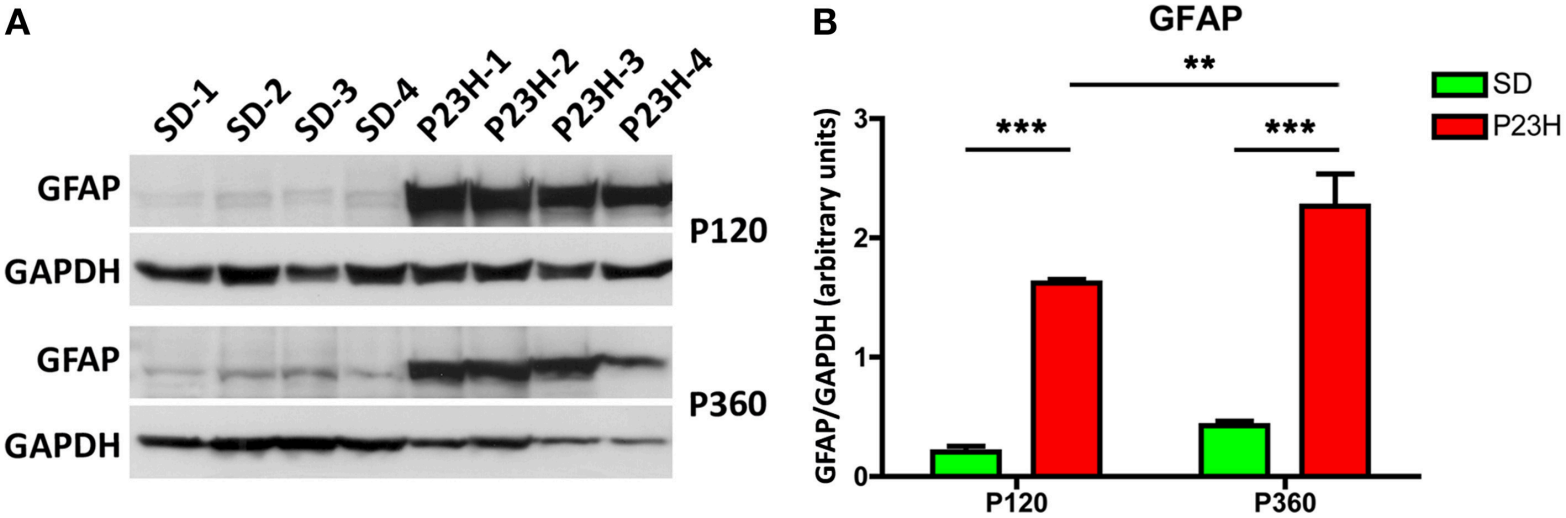
**GFAP expression in SD and P23H rat retinas**. **(A)**Detection of GFAP (apparent molecular weight of 55 kDa) in P23H rat retinas and SD controls by Western blotting at P120 and P360. GAPDH (36 kDa) levels are shown as loading controls. **(B)** The amount of GFAP protein present in each band was densitometrically measured and the value obtained for each sample was normalized to GAPDH levels. Bars represent the average ± SEM (*n* = 4). ^**^P < 0.01; ^***^P < 0.001.

GFAP protein expression was assessed by Western blotting on P23H and SD rat retinas at 4 and 12 months of age (Figures 7A,B). A prominent GFAP band with an estimated SDS-PAGE molecular weight of ~55 kDa was observed in P23H rat retinas compared to SD controls (Figure 7A). Antibodies against GAPDH (36 kDa) were used as loading controls for Western blotting experiments. Thereafter, the amount of GFAP protein present in each band was densitometrically measured and the value obtained for each sample was normalized to GAPDH levels. The normalized expression levels obtained revealed a marked difference of expression between diseased rat retinas and controls (Figure 7B). Expression fold changes were calculated as the ratio between the P23H and control normalized values. Specifically, we observed a 7.8- and 5.3-fold increase in GFAP expression in the retina of P23H rats, as compared to the control animals at the ages of 4 and 12 months, respectively (ANOVA, Bonferroni's test, *P* < 0.001 in both cases). Protein expression analysis also showed significantly greater GFAP levels in P360 vs. P120 P23H rat retinas (ANOVA, Bonferroni's test, *P* < 0.01), indicating that GFAP expression increased in P23H rats during retinal degeneration. This age-related GFAP overexpression in P23H rats was in parallel with the higher GFAP immunoreactivity observed in both astrocytes and Müller cells (Figures 3, 6).

### Expanded Müller cell processes in P23H rat retinas

**Figure 8:**
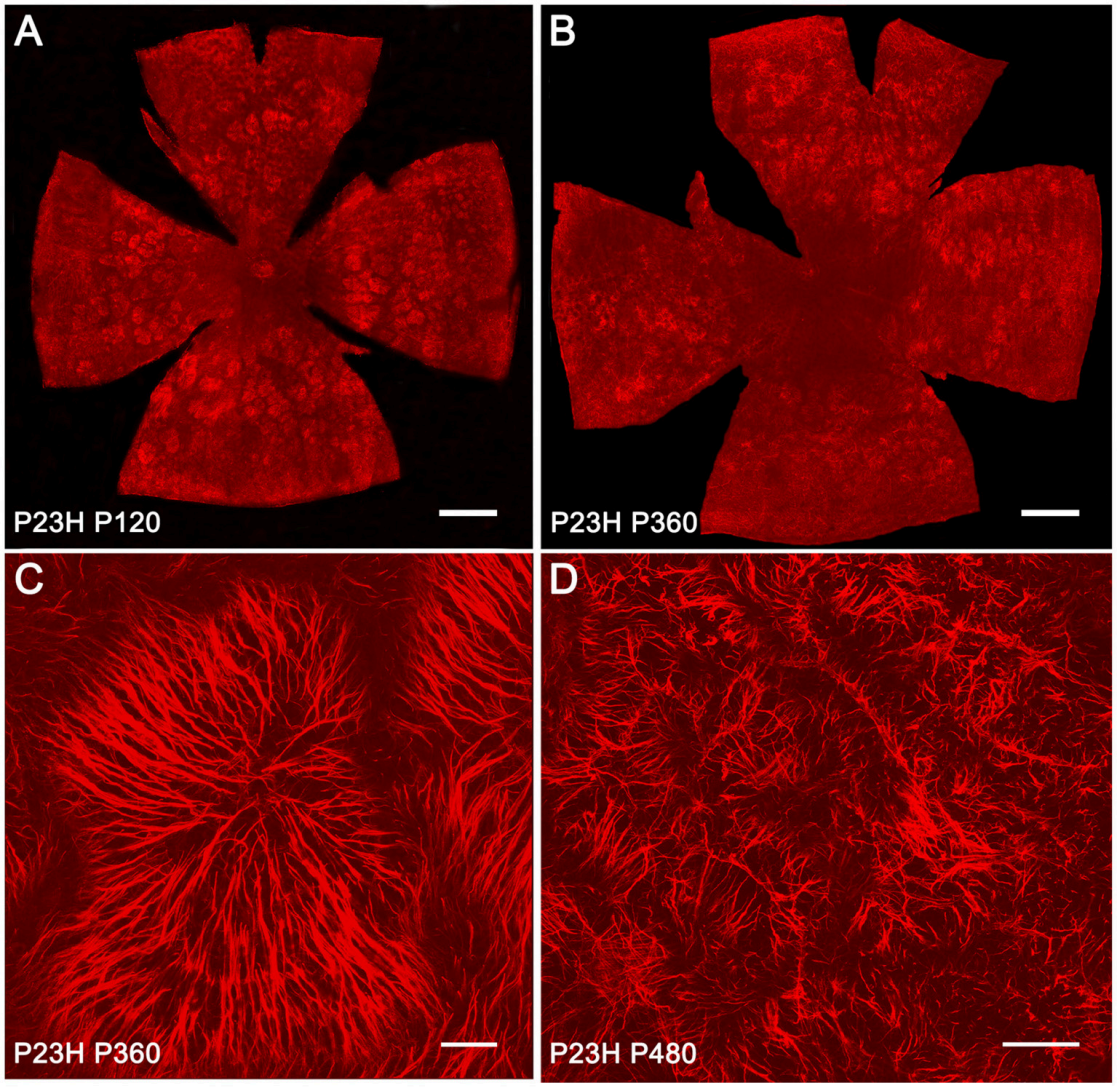
**GFAP-positive Müller cell apical processes in P23H rat retinas**. Whole-mounted retinas from P23H rats at P120 **(A)**, P360 **(B,C**), and P480 **(D)**, showing GFAP-positive Müller cell processes distributed in the ONL. The retinas were flat-mounted with the photoreceptor layer facing up. Note the characteristic firework-like structures generated by Müller cells. Scale bar: 1 mm **(A,B)**, 40 μm **(C)**, 100 μm **(D)**.

Müller cells span across the entire thickness of the neural retina and are essential in maintaining the neuroretinal architecture. In order to assess glial remodeling in degenerative P23H rat retinas, GFAP immunoreactivity was analyzed in the ONL of whole-mounted retinas from SD and P23H rats (Figure 8). In normal retinas, specific GFAP staining was absent in the ONL throughout the retina. However, in diseased retinas, the considerable loss of photoreceptors within the ONL was linked to the appearance of hypertrophied side branches of Müller cells into the outermost photoreceptor layer. At 4 and 12 months of age, apical GFAP-positive Müller cell processes were arranged in clusters, forming characteristic firework-like structures (Figures 8A–C). This peculiar distribution of Müller cell processes in the ONL was not present at 16 months of age (Figure 8D).

**Figure 9:**
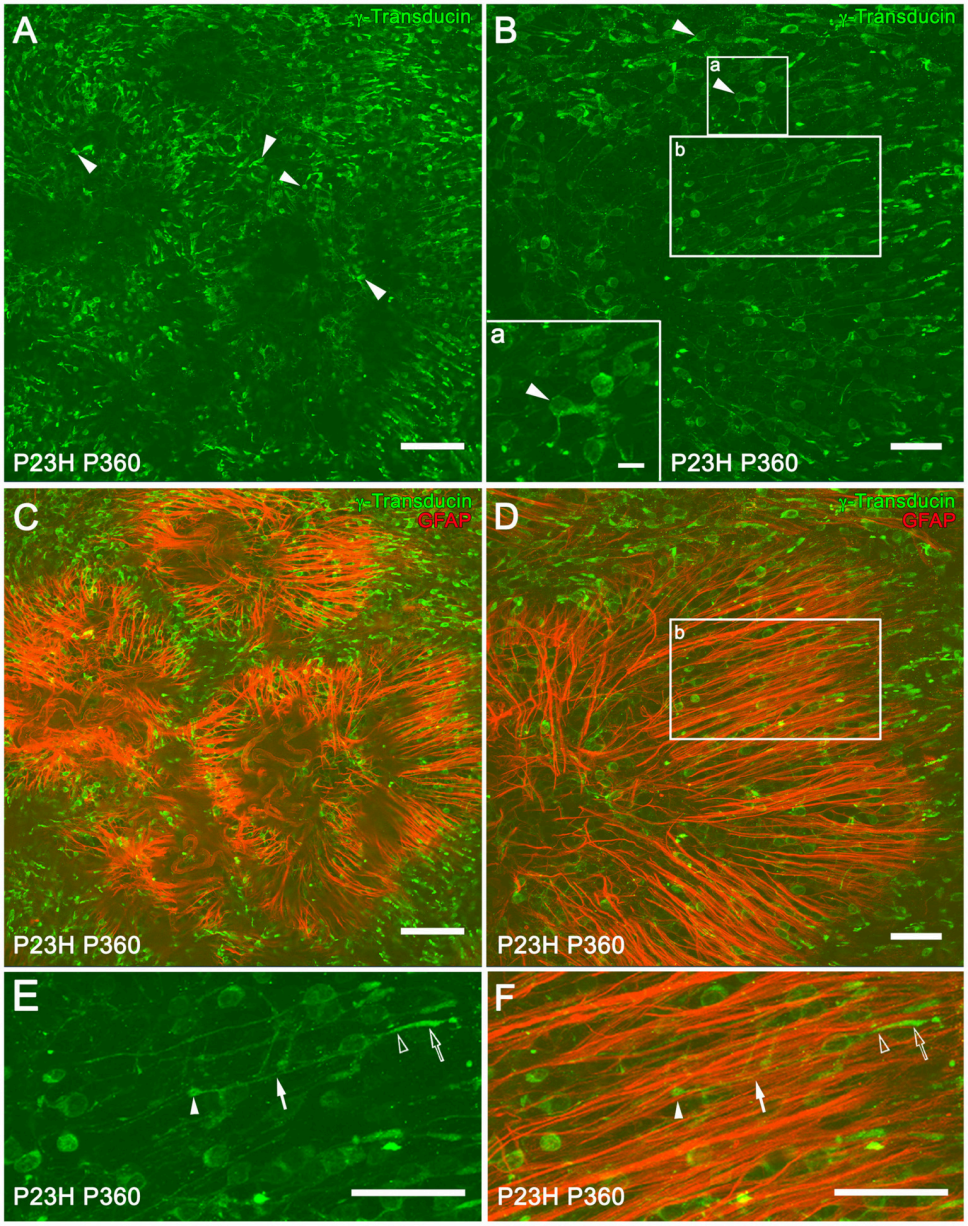
**Müller and cone cell interactions in P23H rat retinas**. Whole-mounted retinas from P360 P23H rats showing single **(A,B)** and double **(C,D)** staining with γ-transducin (green) and GFAP (red) for labeling cone and reactive Müller cells, respectively. The retinas were flat-mounted with the photoreceptor layer facing up. Note the presence of degenerating cones with short outer segments and axons (**A,B**, arrowheads). High magnification of a degenerated cone is shown in **(B)** (lower left corner, inset **a**). **(E,F)** Magnification of **(B,D)** (inset **b**), respectively, showing cones with normal morphology [**E**, outer segment (empty arrow), inner segment (empty arrowhead), axons (arrow), and pedicles (arrowhead)] and Müller cell apical processes (red) arranged in parallel to the cones **(F)**. Scale bar: 100 μm **(A,C)**, 10 μm (Inset **a**), 40 μm **(B,D–F)**.

At 12 months of age in P23H rats, whole-mount retinas showed that most of the cones exhibited a short morphology, indicating a degenerated state (Figures 9A,B, arrowheads). The loss of rod photoreceptors altered the cone mosaic in the ONL. The regular distribution and orientation of cones was disrupted by the appearance of ring-like shaped areas of cone degeneration (Figures 9C,D). A double staining with GFAP and γ-transducin for simultaneous labeling of reactive Müller and cone cells showed a spatial correlation between ring-like shaped areas of cone cells and firework-like structures of Müller cell apical processes in P360 P23H rats (Figures 9B,C). It is interesting to note that some of the cones arranged in parallel on the Müller cell processes showed a normal morphology, with long axons and well-defined outer segments (Figures 9E,F, arrowheads). The morphology of normal and degenerating cones in vertical retinal sections from a SD and P23H rat retinas is shown in Supplementary Figure 1.

## Discussion

The response of the retina to injury results in a set of cell signals that lead to morphological and functional changes, including controlled cell death, and retinal remodeling (Marc et al., 2003; Jones and Marc, 2005; Cuenca et al., 2014). In this context, glial cells play a critical role, and the inflammatory response to injury can be decisive for maintaining the health of the retina or its degeneration (Bringmann et al., 2006; Bringmann and Wiedemann, 2012; Noailles et al., 2014; Hippert et al., 2015). Nevertheless, only a handful of longitudinal studies have considered gliosis in the retina. The current study provides evidence of the common and distinctive features of macroglial changes during retinal degeneration in the P23H rat model of RP.

In P23H rats, retinal astrocytes were activated, proliferated, exhibited enlarged soma and thickened processes, and increased GFAP immunoreactivity. In many mammalian retinas, the density and morphology of astrocytes are strongly influenced by the nearby axons from ganglion cells, and a loss of retinal ganglion cell axons has been linked to a reduced density of astrocytes (Karschin et al., 1986; Gargini et al., 1998). In normal, healthy retinas, astrocytes are densely packed around blood vessels, with larger cells around the veins and smaller ones surrounding the arteries (Jammalamadaka et al., 2015). Here, we showed that retinal astrocyte density in both SD and P23H rats gradually increased from the periphery toward the center of the retina, likely following the center to periphery decrease in retinal neuronal and blood vessel density.

Previous studies have reported that the number of astrocytes in the Wistar rat retina increases between 3 and 9 months and decreases between 9 and 12 months (Mansour et al., 2008). A similar age-related decrease in the number of astrocytes has been observed in the human retina (Ramírez et al., 2001). Our results revealed a slight, progressive increase in astrocyte density in rats at 4, 12, or 16 months of age. On the other hand, in both SD and P23H rats, astrocyte density was significantly lower in adult rats as compared to that observed at P18. This can be attributed, at least partially, to the increase in the relative size of the retina during the postnatal development. The decline in the number of astrocytes from early postnatal age may also be associated with a reduction in the number of retinal ganglion cells during the postnatal development. Previous studies have reported a significant decrease in ganglion cell numbers in the retina of newborn rats during the early weeks of the postnatal period (Perry et al., 1983).

It is assumed that reactive astrogliosis occurs in response to all forms of CNS injury or disease. However, gliotic changes in the degenerating retina may have very different features, depending upon the etiology, severity, and duration of the disease (Ridet et al., 1997; Sofroniew, 2005). Degenerative retinal diseases have been associated with both increases and decreases in astrocyte density. Decreases in the number of astrocytes have been observed in AMD (Ramírez et al., 2001) and during streptozotocin (STZ)-induced diabetes (Ly et al., 2011). The loss of astrocytes is also correlated with the development of pathological retinal neovascularization (Dorrell et al., 2010). On the contrary, no changes or increases in astrocyte density have been described in glaucoma (Wang et al., 2002; Formichella et al., 2014). Our results indicated an increase in astrocyte cell number in P23H rat retinas, as compared to control SD rats at 4 and 12 months of age, when the retina is undergoing a dramatic remodeling as a consequence of retinal degeneration. However, no changes in the number of these cells were observed between SD and P23H at 16 month of age, probably due to the slowdown of the degenerative process in the P23H rat retina from 12 to 16 months. According to this idea, it has been described subtle morphological changes in the P23H line 1 rat retina between 12 and 18 months of degeneration (Pinilla et al., 2015).

The function of astrogliosis is anything but clear, although it has been linked to both protective and harmful effects (Ridet et al., 1997; Sofroniew, 2005). In the acute stage, astrocyte activation is believed to have a beneficial role, boosting the cytoarchitectural remodeling needed to enable glial cells to conserve tissue integrity by restricting the site of the lesion and enhancing their essential neuroprotective function. It has been demonstrated that, after an insult, reactive astrocytes demarcate damaged tissue, restrict inflammation, stimulate the repair of the blood-brain barrier, counteract edema, and protect neurons and neural function (Sofroniew, 2005). Conversely, prolonged activation can interfere with neuronal survival and regeneration.

Using antibodies against Cx43, we observed that Cx43-IR puncta were closely associated with GFAP-IR astrocyte processes at the GCL in both SD and P23H rats. These results are in accordance with previous studies showing Cx43-IR puncta following GFAP-IR astrocyte processes on the inner surface of the rat retina (Kerr et al., 2010), and imply that the gap junctions formed by retinal astrocytes contain Cx43. Cx43-IR was previously detected in the GCL of rat retinas (Janssen-Bienhold et al., 1998). In humans, Cx43-IR has been found on GFAP-positive astrocytes in the retinal GCL and on the optic nerve head (Kerr et al., 2011). In P23H rat retinas, the density of Cx43-IR puncta within the GCL and the GFAP protein expression were greater than in SD rat retinas. Increased Cx43 expression has been previously described following acute injury to the CNS (Lee et al., 2005; Nakase et al., 2006; Haupt et al., 2007) and in neurodegenerative diseases (Rufer et al., 1996; Vis et al., 1998). In glaucomatous eyes, increased Cx43-IR was observed in the peripapillary and mid-peripheral retina, in association with glial activation (Kerr et al., 2011). These observations may suggest a role for Cx43 in the pathogenesis of neurodegeneration. The higher expression of Cx43 and the increase in Cx43-IR puncta observed in P23H rats were in correlation with the astrocyte cell hypertrophy observed in these animals. The main features of this hypertrophy are the elongation and proliferation of astrocyte processes, as well as the swelling of both cell bodies and processes, which is reflected in the complexity of the astrocytic network. In this context, because the number of astrocytes increased 20% and 23% in P120 and P360 P23H rat retinas, respectively, as compared to control animals and the increase in Cx43 expression levels detected by Western blot was much higher (125 and 82% respectively), we suggest an increment of Cx43 expression per astrocyte. The overall increase in Cx43 could be the consequence of both the increased number of astrocytes and of their complexity in order to maintain the communication between adjacent cells throughout gap-junctions.

It is assumed that retinal diseases and injuries upregulate the GFAP, as well as additional intermediate filament proteins; this is thought to occur primarily in the Müller cells (Fisher and Lewis, 2003; Bringmann et al., 2006; Bringmann and Wiedemann, 2012). Thus, a common feature of all retinal diseases, including RP, glaucoma, DR, and AMD in both animal models and humans, is the presence of a high level of GFAP in Müller cells (Madigan et al., 1994; Strettoi et al., 2002; Wang et al., 2002; Gerhardinger et al., 2005; Inman and Horner, 2007; Vogler et al., 2014; Hippert et al., 2015). This effect has been related to modifications in a potassium channel referred to as KCNQ5, suggesting a relationship between cytoskeletal proteins such as GFAP, and membrane proteins, such as potassium channels (Caminos et al., 2015). In addition to the increase in intermediate filament proteins, reactive Müller cells may become hypertrophied, resulting in a proliferation of fibrous processes on the outer edge of the retina (Fisher and Lewis, 2003; Marc and Jones, 2003; Gargini et al., 2007; Ray et al., 2010; Cuenca et al., 2014). The expanded Müller cell processes occupy the space left by the dying photoreceptors. We observed that GFAP immunoreactivity within Müller cells was markedly greater in P23H rats than that observed in age-matched control rats. In normal retinas, GFAP immunoreactivity was not detected, while diseased retinas showed intense GFAP immunoreactivity within the entire cell, from their end-feet to the outer limiting membrane. Moreover, hypertrophied branches of Müller cells were observed into the ONL of diseased retinas.

In parallel with the immunohistochemistry data, Western blotting results showed a large increase in GFAP protein expression in the retina of P23H rats, as compared to the control animals. This increment reflects GFAP expression in the activated Müller cells. However, it is important to note that the astrocytic hyperplasia and hypertrophy observed in the degenerative retina of P23H rats also contribute to the upregulation of GFAP expression. Additionally, in P23H rat retinas, GFAP expression also increases with the age of the animal, indicating enhanced activation of glial cells in the advanced stages of retinal degeneration. The function of increased GFAP expression in Müller cells is unknown, although is thought to help stabilize the newly formed terminal processes of Müller cells and provide resistance to mechanical stress (Lundkvist et al., 2004; Verardo et al., 2008). Furthermore, increased levels of GFAP would seem to be essential for a multitude of responses during Müller cell gliosis, such as the formation of glial scars, neurite growth, infiltration of monocytes, neovascularization, and the integration of cells in retinal transplants, as all these characteristics were found to be attenuated in GFAP- and vimentin-deficient mice, which are an experimental animal model of retinal detachment (Lewis and Fisher, 2003; Nakazawa et al., 2007; Bringmann et al., 2009).

The cytoarchitecture of the outer nuclear layer is, at least in part, maintained by a network of heterotypic adherens junction complexes that form the outer limiting membrane (OLM) (Williams et al., 1990). These junctions connect the inner segments of photoreceptors to the apical processes of Müller cells, and thus photoreceptor death is likely to disrupt the OLM, and compromise the orientation and polarity of the photoreceptors (Hippert et al., 2015). In this study, the regular distribution and orientation of cone cells was disrupted by the appearance of ring-shaped areas of cone degeneration, with Müller cell apical processes forming clusters that resembled fireworks. This peculiar disposition of cones and apical Müller cell processes has been previously described in the S334ter-line-3 rat (Lee et al., 2011; Ji et al., 2015) and the P23H line 1 rat (García-Ayuso et al., 2013) models of RP. In agreement with previous studies (Cuenca et al., 2004, 2014; Lu et al., 2013), as photoreceptor degeneration progressed, cone morphology underwent progressive changes, including the shortening of both outer segments and axons. However, our results show for the first time that Müller cell apical processes are arranged parallel to some cones, which retain normal morphology in advanced stages of retinal degeneration. We also show that the spatial correlation between ring-like shaped areas of cone cells and firework-like structures of Müller cell apical processes are transient structures that disappear in late stages of retinal degeneration.

One can hypothesize that the loss of photoreceptors in this RP model, the P23H rat, induces changes in retinal vasculature (Villegas-Pérez et al., 1998; Pennesi et al., 2008), which, in turn, may induce the activation of astrocytes and Müller cells. However, given that retinal macroglia is essential for the maintenance of retinal homeostasis, morphological changes, and activation of astrocytes and Müller cells can induce inner retinal remodeling (Marc et al., 2003; Cuenca et al., 2004, 2014; Jones and Marc, 2005; Puthussery and Taylor, 2010; Jones et al., 2012), including ganglion cell degeneration (Jones et al., 2003; García-Ayuso et al., 2010; Kolomiets et al., 2010). The disruption of blood vessels can affect the normal oxygen and nutrient supply to retinal cells, and thereby accelerate the progress of retinal degeneration. Most likely, inner retinal remodeling is mediated by changes in both retinal vasculature and macroglial cells after photoreceptor death in retinitis pigmentosa.

## Author contributions

Conception and design of the work: NC. Data acquisition and analysis: LF, LC. Data interpretation: LF, PL, IP, LC, NC. Drafting of the manuscript: LF, LC, PL, IP, NC.

### Conflict of interest statement

The authors declare that the research was conducted in the absence of any commercial or financial relationships that could be construed as a potential conflict of interest.

## Supplementary material

Supplementary Figure 1 **Cone cells in SD and P23H rat retinas**. Vertical retinal sections from a SD rat **(A)** and a P23H rat at P120 stained with γ-transducin, showing the morphology of normal cones in SD rats and degenerating cones in P23H rats. Note the typical cone shape in SD rats, where outer (empty arrows) and inner segments (empty arrowheads), axons (arrows) and pedicles (arrowheads) are clearly recognizable. By contrast, in P23H rat retinas the cone outer segments are both short and swollen and very small in size, the axons are nor identifiable and pedicles seem to emerge directly from the cone cell bodies. Both images were collected from the central area of the retina, close to the optic nerve. Scale bar: 20 μm.
