# Supplementary figures and images for: Astrocytes and Müller Cell Alterations During Retinal Degeneration in a Transgenic Rat Model of Retinitis Pigmentosa
Source: Front Cell Neurosci. 2015 Dec 22;9:484. doi: 10.3389/fncel.2015.00484 (PMC4686678; doi:10.3389/fncel.2015.00484)

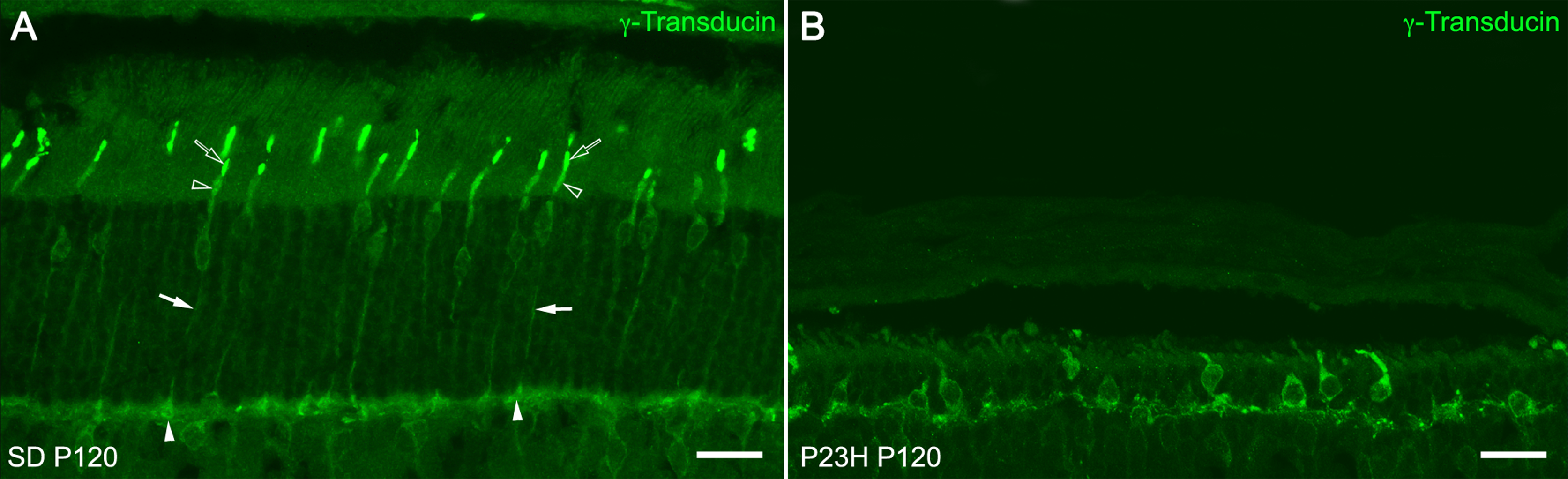

Supplement: Supplementary Figure 1 — Cone cells in SD and P23H rat retinas. Vertical retinal sections from a SD rat (A) and a P23H rat at P120 stained with γ-transducin, showing the morphology of normal cones in SD rats and degenerating cones in P23H rats. Note the typical cone shape in SD rats, where outer (empty arrows) and inner segments (empty arrowheads), axons (arrows) and pedicles (arrowheads) are clearly recognizable. By contrast, in P23H rat retinas the cone outer segments are both short and swollen and very small in size, the axons are nor identifiable and pedicles seem to emerge directly from the cone cell bodies. Both images were collected from the central area of the retina, close to the optic nerve. Scale bar: 20 μm. [file Image1.TIF]
